# Association Between Serum Levels of Vitamin D and the Risk of Post-Stroke Anxiety

**DOI:** 10.1097/MD.0000000000003566

**Published:** 2016-05-06

**Authors:** Chaowen Wu, Wenwei Ren, Jianhua Cheng, Beilei Zhu, Qianqian Jin, Liping Wang, Cao Chen, Lin Zhu, Yaling Chang, Yingying Gu, Jiyun Zhao, Dezhao Lv, Bei Shao, Shunkai Zhang, Jincai He

**Affiliations:** From the Department of Neurology (CW, WR, JC, BZ, QJ, LW, CC, LZ, YC, YG, JZ, DL, BS, JH), The First Affiliated Hospital of Wenzhou Medical University and Department of Neurology, Ruian People's Hospital, Wenzhou (SZ), People's Republic of China.

## Abstract

Low levels of serum vitamin D are common in patients with mood disorders and stroke. It has been shown that low levels of serum vitamin D indicate a risk of depression in post-stroke subjects. Our aim was to determine the relationship between vitamin D and post-stroke anxiety (PSA).

A consecutive series of 226 first acute ischemic stroke patients were recruited and followed up for 1 month. Serum levels of vitamin D were measured within 24 hours of admission. Patients with significant clinical symptoms of anxiety and a Hamilton anxiety scale score >7 were diagnosed as having PSA. In addition, 100 healthy subjects were recruited as controls and underwent measurements of serum vitamin D.

A total of 60 patients (26.55%) showed anxiety at 1 month. Both PSA patients and non-PSA patients had lower serum levels of vitamin D than healthy subjects. A significant relationship was found between PSA and serum levels of vitamin D. Low serum levels of vitamin D (≤38.48 nmol/L) were independently associated with the development of PSA (OR: 2.49, 95% CI: 1.21–5.13, *P* = 0.01).

Serum vitamin D status is related to the occurrence of anxiety in post-stroke patients and may be an independent risk factor of PSA after 1 month.

## INTRODUCTION

Mental disorders such as anxiety and depression are a particularly frequent and important complication of stroke. Compared with post-stroke depression, anxiety after stroke has been relatively neglected. Previous studies have found that approximately a quarter of stroke patients experience post-stroke anxiety (PSA).^1,2^ Several studies have also noted that 21% of patients suffered from moderate or severe anxiety 3 months after a stroke,^3^ whereas the frequency of PSA was 18% at 2 years.^4^ Many factors have been demonstrated to be correlated with the development of PSA, including gender,^4,5^ age,^4^ lesion location,^6,7^ severity of cognitive impairment,^3,8^ and activities of daily living.^4^ Previous studies have strongly suggested that symptoms of anxiety negatively influence rehabilitation and long-term outcomes.^9,10^ Therefore, early recognition and treatment of PSA is of great importance to reduce stroke complications and mortality as well as to improve functional outcomes. However, the underlying pathophysiological mechanisms of the development of PSA remain unclear.

Vitamin D has been proposed to be a neurosteroid hormone in the central nervous system and plays an important role in the development of the brain. Vitamin D receptor (VDR) and tryptophan hydroxylase, which are required for the synthesis of the active metabolite, are widely present in the brain.^11,12^ Vitamin D plays diverse biological roles in certain mental processes, such as enabling neuroprotection,^12^ regulating neurotrophic signaling,^13^ and influencing inflammation.^14,15^ Previous studies have suggested that a deficiency in Vitamin D is linked with a wide range of nervous system diseases, including Alzheimer disease,^16^ Parkinson disease,^16^ and multiple sclerosis.^17^ Multiple lines of evidence have indicated that low serum levels of vitamin D are prevalent in the majority of acute stroke patients^18,19^ and can be regarded as a potential risk factor for stroke.^19,20^ Furthermore, it has been reported that low serum concentrations of vitamin D in stroke patients might contribute to a higher morbidity and mortality.^21^ Additionally, vitamin D supplementation has been found to decrease the incidence of stroke and improve the outcome of patients.^19,21^ There is a growing body of evidence suggesting a strong relationship between vitamin D deficiency and emotional disorders including depression and anxiety.^22,23^ A large cross-sectional study revealed that higher vitamin D levels could significantly reduce the risk of depression in healthy populations.^24^ A relationship between lower levels of vitamin D and depression have been documented in depressed patients.^25^ Given the similar neural pathways to depression, low levels of vitamin D have also been found in patients with anxiety disorder.^26^ Moreover, animal experiments have demonstrated that mice with a vitamin D deficiency showed an increase in anxiety-like behaviors.

Vitamin D deficiency is very common in stroke patients and has been related to the development of post-stroke depression.^27,28^ Nevertheless, the effects of vitamin D on the incidence of PSA remain unclear. Therefore, we conducted this study to explore the potential association between serum levels of vitamin D and the development of PSA.

## METHODS

### Subjects

This research was conducted in the First Affiliated Hospital of Wenzhou Medical University. Patients within 7 days of stroke onset who were between the ages of 18 and 80 years old were recruited from October 2013 to September 2014. The diagnosis of acute ischemic stroke was supported by computed tomography scanning and/or magnetic resonance imaging. The exclusion criteria included patients with the following characteristics: (1) a decreased level of consciousness or severe cognitive dysfunction, aphasia, or dysarthria; (2) a history of anxiety disorder or other psychiatric disorder; (3) a history of stroke or any central nervous system disease such as Parkinson disease, dementia, or a tumor; and (4) the presence of severe physical diseases that resulted in an inability to follow up. Every patient signed an informed consent form. A total of 100 healthy subjects with a similar age and gender were recruited as controls. This study followed ethical guidelines and obtained the approval of the Institutional Review Board of the First Affiliated Hospital of Wenzhou Medical University.

### Clinical Characteristics

All demographics characteristics including age, gender, marital status, educational status, and economic status were collected at baseline. Stroke severity was assessed by the National Institutes of Health Stroke Scale (NIHSS) at admission and 1 month. Stroke outcomes were measured by the modified Rankin scale (mRS) and the Barthel index (BI), and cognition function was assessed by the mini-mental state examination (MMSE) at 1 month.

All of the patients were screened for anxiety symptoms using the Hamilton anxiety scale (HAMA) at 1 month. Subjects with a HAMA anxiety score >7 were considered to have existing symptoms of anxiety.

### Measurement of Vitamin D

Blood samples were collected using tubes with ethylene diamine tetra-acetic acid anticoagulant and were centrifuged to collect patient serum within 24 hours of admission. Because of its widespread clinical application and standardized ranges, we chose serum 25-hydroxyvitamin D [25(OH)D] as the index of vitamin D status for all of the subjects. Serum levels of [25(OH)D] were measured through a competitive protein-binding assay, and the intra-assay coefficient of variation was 7% to 10%. Serum vitamin D data were divided into 4 quartiles because of its skewed distribution (≤38.48, 38.48–52.42, 52.42–63.18, and ≥63.18 nmol/L).

### Statistical Analysis

The Mann–Whitney *U* test, Student *t* test, or χ^2^ test were appropriately used to determine the differences between groups. Nonlinear variables were performed with logit-transformation for linear distributions. Logistic regression was employed to analyze independent risk factors of PSA. All statistical analyses were performed using SPSS for Windows (Release 19.0; SPSS, Chicago, IL). A *P*-value <0.05 was considered statistically significant.

## RESULTS

In this study, a total of 552 first acute ischemic stroke patients were screened, with 226 finally enrolled. The mean age was 63.13 years, and 37.16% were women. Sixty cases showed anxiety, and the incidence of PSA was 26.55% at 1 month after stroke. The background characteristics of the participants are shown in Table 1. We did not find significant differences between PSA and non-PSA patients in age (*P* = 0.81), sex (M/F) (*P* = 0.25), body mass index (BMI) (*P* = 0.38), or education (*P* = 0.26). Compared with the non-PSA group, the PSA group had more severe stroke (NIHSS score 2(0–12) vs 3(0–14), *P* = 0.02), poorer cognitive function (MMSE score 26(11–30) vs 24(10–30), *P* = 0.04), worse functional outcome (mRS score 1(0–4) vs 3(0–4), *P* <0.001), and poorer activities of daily living (BI score 100(30–100) vs 95(30–100), *P* <0.001) (Table 1).

**TABLE 1:**
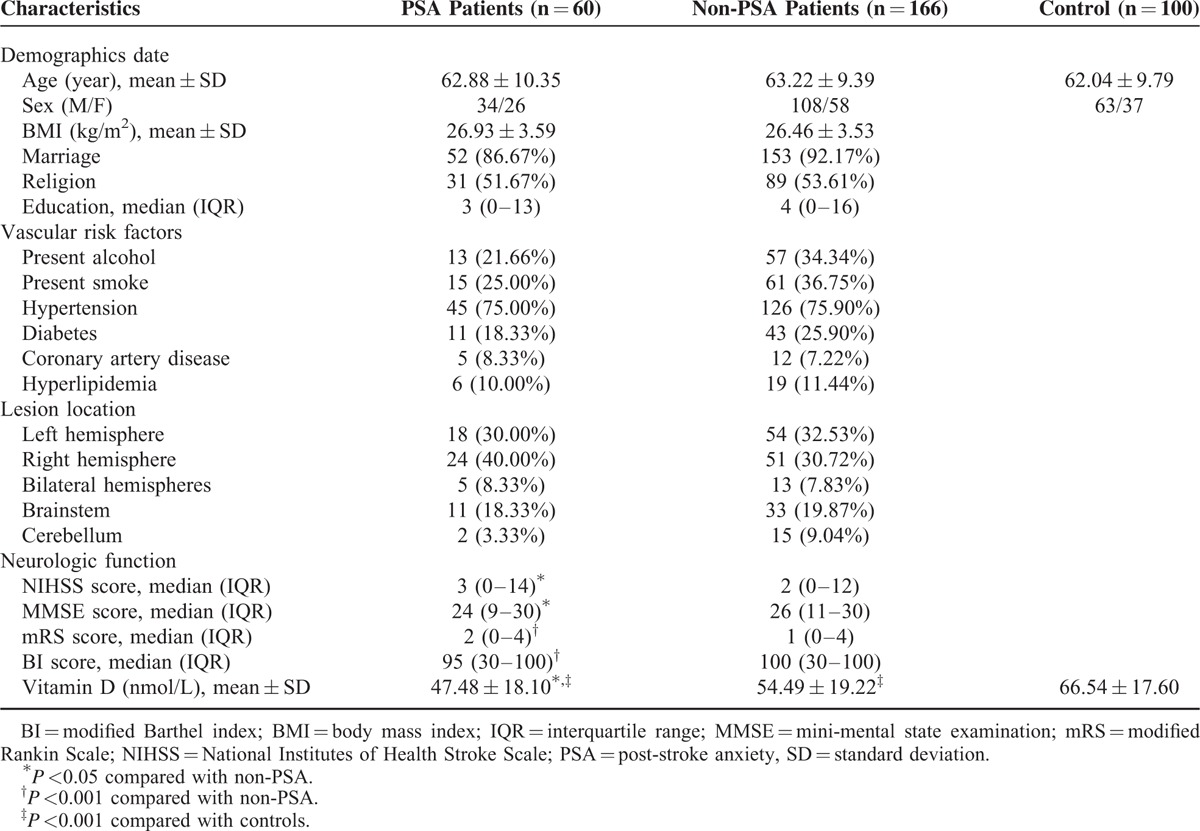
Clinical Characteristics of the Study Population

The mean level of serum vitamin D in stroke patients was 52.63 ± 19.14, which was much lower than that of normal subjects (66.54 ± 17.57, *P* <0.001). Serum vitamin D was found to be significantly lower in the PSA group than in the non-PSA group (47.48 ± 18.10 vs 54.49 ± 19.22, respectively, *P* = 0.02). Moreover, the serum vitamin D of both these two groups was lower than that of controls. Next, we divided patients into four groups according to quartiles of serum vitamin D levels, and we found significant differences in patients in the lowest quartile (*P* = 0.01) (Table 2).

**TABLE 2:**
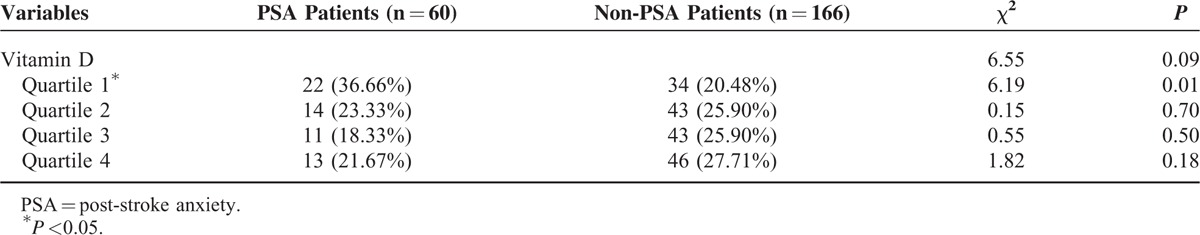
Vitamin D Level Quartiles of Subjects

With the last three quartiles of vitamin D levels used as the reference and the occurrence of PSA considered the dependent variable in the logistic analysis, serum concentration of vitamin D (≤38.48 nmol/L) were independently associated with an increased risk of PSA (odds ratios (OR) 2.49, 95% confidence interval (CI): 1.21–5.13, *P* = 0.01) after adjusting for possible confounders. In addition, the MMSE scores at 1 month were significantly associated with the occurrence of PSA in first acute ischemic stroke patients (OR 0.92, 95% CI: 0.86–0.99, *P* = 0.02) (Table 3).

**TABLE 3:**
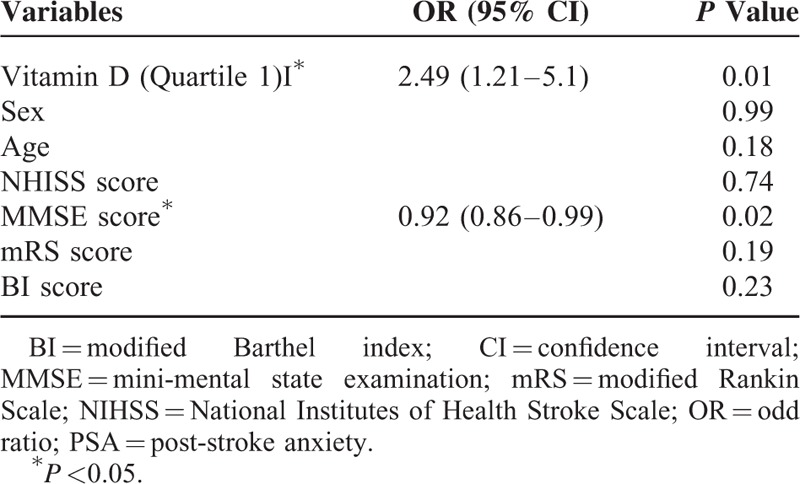
Multivariate Logistic Model of the Clinical Determinants of PSA

## DISCUSSION

To the best of our knowledge, this is the first study to explore the relationship between serum vitamin D levels and the occurrence of PSA. Our results revealed an inverse association between serum vitamin D levels and anxiety 1 month after stroke.

Previous studies have demonstrated that ∼11% to 54% of stroke patients experience anxiety symptoms,^3,8,29–32^ which was similar to our results. A meta-analysis of 41 studies reported a pooled PSA prevalence of 18% in the acute period, without finding a significant increase over time.^1^ A 10-year follow-up study indicated that PSA was a common problem in a long-term observation, with a prevalence over 30% and an annual incidence of ∼20%.^33^

As a common and long-lasting complication, early recognition and treatment are particularly important, but the underlying mechanism of PSA has remained unclear. In our study, the serum concentration of vitamin D was found to be significantly lower in acute stroke patients than in healthy controls, which was consistent with previous studies.^18,19^ Moreover, a significant association between low serum levels of vitamin D and PSA was also found. This suggests that serum levels of vitamin D might be used as a biological marker of PSA.

Over the past decades, there have been a growing number of studies exploring the relationship between vitamin D and anxiety.^22,23^ Vitamin D could modulate anxiety symptoms through its effect on inflammation, as mediated by cytokines.^14^ An increased level of C-reaction protein has been found in men with present anxiety disorder,^34^ whereas the serum levels of vitamin D have been negatively correlated with various cytokines such as TNF-α and C-reaction protein.^15^ Additionally, cytokines could lead to anxiety by altering the metabolism of neurotransmitters such as dopamine^35^ and serotonin.^36^ Interferon-alpha was found to downregulate glucocorticoid receptor and serotonin receptor 1A levels in cell lines,^36^ which was believed to be closely connected with anxiety. Moreover, interferon-gamma knockout mice exhibited anxiogenic behavior.^37^ The function of the hypothalamic–pituitary–adrenal (HPA) axis, which has been confirmed to be another plausible mechanism involved in the process of anxiety, is also influenced by cytokines.^38^ A previous study demonstrated that interferon and tumor necrosis factor alpha (TNF-α) may contribute to altered diurnal HPA axis activity.^39^

Multiple lines of evidence have suggested that vitamin D deficiency is prevalent in post-stroke patients because of decreased vitamin D intake and sunlight caused by limited mobility.^18,19^ The incidence of vitamin D deficiency in post-stroke patients was found to be greater than that in other inpatients with general medical disease.^40^ As a potent anti-inflammatory agent, vitamin D might be involved in the development of PSA through its influence on the expression of cytokines and its mediation of inflammation.^24^ A variety of cells in the brain including neurons, astrocytes, and microglia release bioactive cytokines.^25^ Acute ischemic stroke can modulate the expression of cytokines in these cells.^41^ Specific cytokines including interleukin-1 and TNF-α have been heavily implicated in acute stroke patients,^27,42^ and similar results have also been observed in ischemic brains of aged mice.^28^ Vitamin D could reduce brain inflammation after brain injury.^43^

In addition to the inflammation mentioned previously, vitamin D might also lead to PSA through its effect on the synthesis of neurotrophins. Vitamin D has been shown to regulate neurotrophic signaling.^44^ The expression of several neurotrophic factors such as nerve growth factor, neurotrophin-3, and neurotrophin-4 has been found to be influenced by calcitriol (the primary active form of vitamin D), enhancing transmission of synaptic or regulatory calcium signaling.^45–47^ Studies in animals have identified that serum levels of nerve growth factor are related to inducing anxiolytic-like behaviors in mice.^48^

Furthermore, vitamin D mediates its function via binding to VRD and the enzyme 1a-hydroxylase, which are widely located in neuronal and glial cells of the human brain.^49^ Previous studies have found that VDR knock-out mice showed increased anxiety symptoms.^50^ Therefore, it was speculated that defects in the vitamin D-VDR system may directly result in anxiety. Hence, we can propose that low serum levels of vitamin D might play a critical role in the development of PSA.

Our results suggested that cognitive function decline after stroke was a risk factor for the development of PSA, which is broadly consistent with the findings of previous studies.^8^ Meanwhile, no relationship was found between the development of PSA and other variables, including severity of stroke,^51^ being female,^4,5^ and lesion location.^6,7^

There were several limitations to the present study. First, the serum level of vitamin D was tested only at admission, and thus further studies are required to explore how serum levels of vitamin D change dynamically after a stroke. Second, patients with severe cognitive dysfunction and aphasia were excluded, which may have introduced bias into our study. Third, the duration of follow-up might not have been long enough for us to distinguish “true” anxiety from “reactive” anxiety. Finally, the role of vitamin D-binding protein in anxiety was not studied. Studies with large samples and a long-term follow-up are needed to explore the relationship between vitamin D and PSA.

## CONCLUSION

In summary, our study demonstrates that serum levels of vitamin D at admission were associated with the development of PSA. Moreover, vitamin D deficiency was considered an independent risk factor for PSA at 1 month. Further prospective studies are necessary to confirm this association and may contribute to the prevention and treatment of PSA.

## References

[1] Campbell Burton CA Murray J Holmes J Frequency of anxiety after stroke: a systematic review and meta-analysis of observational studies. *Int J Stroke* 2013; 8:545–559. 23013268 10.1111/j.1747-4949.2012.00906.x

[2] Menlove L Crayton E Kneebone I Predictors of anxiety after stroke: a systematic review of observational studies. *J Stroke Cerebrovasc Dis* 2015; 24:1107–1117. 25816724 10.1016/j.jstrokecerebrovasdis.2014.12.036

[3] Barker-Collo SL Depression and anxiety 3 months post stroke: prevalence and correlates. *Arch Clin Neuropsychol* 2007; 22:519–531. 17462857 10.1016/j.acn.2007.03.002

[4] Schultz SK Castillo CS Rosier JT Generalized anxiety and depression: assessment over 2 years after stroke. *Am J Geriatr Psychiatry* 1997; 5:229–237. 9209565 10.1097/00019442-199700530-00007

[5] MVJMMW R Predictors of distress following an acute stroke: disability, control cognitions, and satisfaction with care. *Psychol Health* 2000; 15:395.

[6] C-PFH-PJZMR-FBH-BJG-FCC-RBC-P JA Influence of premorbid psychopathology and lesion location on affective and behavioral disorders after ischemic stroke. *J Neuropsychiatry Clin Neurosci* 2003; 23:340–347. 10.1176/jnp.23.3.jnp340 21948896

[7] Tang WK Chen Y Lu J Frontal infarcts and anxiety in stroke. *Stroke* 2012; 43:1426–1428. 22282881 10.1161/STROKEAHA.111.640482

[8] Fure B Wyller TB Engedal K Emotional symptoms in acute ischemic stroke. *Int J Geriatr psychiatry* 2006; 21:382–387. 16534769 10.1002/gps.1482

[9] van Mierlo ML Schroder C van Heugten CM The influence of psychological factors on health-related quality of life after stroke: a systematic review. *Int J Stroke* 2014; 9:341–348. 24148550 10.1111/ijs.12149

[10] Shimoda K Robinson RG Effects of anxiety disorder on impairment and recovery from stroke. *J Neuropsychiatry Clin Neurosci Winter* 1998; 10:34–40. 10.1176/jnp.10.1.34 9547464

[11] Kesby JP Eyles DW Burne THJ The effects of vitamin D on brain development and adult brain function. *Mol Cell Endocrinol* 2011; 347:121–127. 21664231 10.1016/j.mce.2011.05.014

[12] Kalueff AVT Pentti Neurosteroid hormone vitamin D and its utility in clinical nutrition. *Curr Opin Clin Nut Metab Care* 2007; 10:12–19. 10.1097/MCO.0b013e328010ca18 17143049

[13] Dicou E Neurotrophins and neuronal migration in the developing rodent brain. *Brain Res Rev* 2009; 60:408–417. 19306897 10.1016/j.brainresrev.2009.03.001

[14] Borges MC Martini LA Rogero MM Current perspectives on vitamin D, immune system, and chronic diseases. *Nutrition* 2011; 27:399–404. 20971616 10.1016/j.nut.2010.07.022

[15] Bellia A Garcovich C D’Adamo M Serum 25-hydroxyvitamin D levels are inversely associated with systemic inflammation in severe obese subjects. *Internal and emergency medicine* 2013; 8:33–40. 21437585 10.1007/s11739-011-0559-x

[16] Evatt ML Delong MR Khazai N Prevalence of vitamin d insufficiency in patients with Parkinson disease and Alzheimer disease. *Arch Neurol* 2008; 65:1348–1352. 18852350 10.1001/archneur.65.10.1348 PMC2746037

[17] Hayesa CE Vitamin D: a natural inhibitor of multiple sclerosis. *Proc Nut Soc* 2000; 59:531–535. 10.1017/s0029665100000768 11115787

[18] Poole KE Loveridge N Barker PJ Reduced vitamin D in acute stroke. *Stroke* 2006; 37:243–245. 16322500 10.1161/01.STR.0000195184.24297.c1

[19] Stefania E Makariou PM Meropi S Vitamin D and stroke: promise for prevention and better outcome. *Curr Vasc Pharmacol* 2014; 12:117–124. 22724468 10.2174/15701611113119990119

[20] Grant WB Epidemiology of disease risks in relation to vitamin D insufficiency. *Prog Biophys Mol Biol* 2006; 92:65–79. 16546242 10.1016/j.pbiomolbio.2006.02.013

[21] BJD-HBSTWDDGQWBPRIFMPSBR.T KL 25-Hydroxyvitamin D, dementia, and cerebrovascular pathology in elders receiving home services. *Neurology* 2010; 74:18–26. 19940273 10.1212/WNL.0b013e3181beecb7 PMC2809024

[22] Hogberg G Gustafsson SA Hallstrom T Depressed adolescents in a case-series were low in vitamin D and depression was ameliorated by vitamin D supplementation. *Acta Paediatrica* 2012; 101:779–783. 22372707 10.1111/j.1651-2227.2012.02655.x

[23] May HT Bair TL Lappe DL Association of vitamin D levels with incident depression among a general cardiovascular population. *Am Heart J* 2010; 159:1037–1043. 20569717 10.1016/j.ahj.2010.03.017

[24] Griffin MD Xing N Kumar R Vitamin D and its analogs as regulators of immune activation and antigen presentation. *Ann Rev Nutr* 2003; 23:117–145. 12651965 10.1146/annurev.nutr.23.011702.073114

[25] Leonard BE Myint A The psychoneuroimmunology of depression. *Human Psychopharmacol* 2009; 24:165–175. 10.1002/hup.1011 19212943

[26] Armstrong DJ Meenagh GK Bickle I Vitamin D deficiency is associated with anxiety and depression in fibromyalgia. *Clin Rheumatol* 2007; 26:551–554. 16850115 10.1007/s10067-006-0348-5

[27] Szczepanska-Szerej A Kurzepa J Wojczal J Simvastatin-induced prevention of the increase in TNF-alpha level in the acute phase of ischemic stroke. *Pharmacol Rep* 2007; 59:94–97. 17377212

[28] Shin JA Jeong SI Kim M Visceral adipose tissue inflammation is associated with age-related brain changes and ischemic brain damage in aged mice. *Brain Behav Immun* 2015; 50:221–231. 26184082 10.1016/j.bbi.2015.07.008

[29] Merriman C Norman P Barton J Psychological correlates of PTSD symptoms following stroke. *Psychol Health Med* 2007; 12:592–602. 17828679 10.1080/13548500601162747

[30] Field EL Norman P Barton J Cross-sectional and prospective associations between cognitive appraisals and posttraumatic stress disorder symptoms following stroke. *Behav Res Ther* 2008; 46:62–70. 18005937 10.1016/j.brat.2007.10.006

[31] Lincoln NB Brinkmann N Cunningham S Anxiety and depression after stroke: a 5 year follow-up. *Disabil Rehabil* 2013; 35:140–145. 22725629 10.3109/09638288.2012.691939

[32] Sagen U Finset A Moum T Early detection of patients at risk for anxiety, depression and apathy after stroke. *Gen Hosp Psychiatry* 2010; 32:80–85. 20114132 10.1016/j.genhosppsych.2009.10.001

[33] Ayerbe L Ayis SA Crichton S Natural history, predictors and associated outcomes of anxiety up to 10 years after stroke: the South London Stroke Register. *Age Ageing* 2014; 43:542–547. 24375225 10.1093/ageing/aft208

[34] Vogelzangs N Beekman AT de Jonge P Anxiety disorders and inflammation in a large adult cohort. *Translational Psychiatry* 2013; 3:e249. 23612048 10.1038/tp.2013.27 PMC3641413

[35] Moron JA Zakharova I Ferrer JV Mitogen-activated protein kinase regulates dopamine transporter surface expression and dopamine transport capacity. *J Neurosc* 2003; 23:8480–8488. 10.1523/JNEUROSCI.23-24-08480.2003 PMC6740378 13679416

[36] Cai W Khaoustov VI Xie Q Interferon-alpha-induced modulation of glucocorticoid and serotonin receptors as a mechanism of depression. *J Hepatol* 2005; 42:880–887. 15885359 10.1016/j.jhep.2005.01.024

[37] Campos AC Vaz GN Saito VM Further evidence for the role of interferon-gamma on anxiety- and depressive-like behaviors: involvement of hippocampal neurogenesis and NGF production. *Neurosc Lett* 2014; 578:100–105. 10.1016/j.neulet.2014.06.039 24993299

[38] Pariante CM Miller AH Glucocorticoid receptors in major depression: relevance to pathophysiology and treatment. *Biol Psychiatry* 2001; 49:391–404. 11274650 10.1016/s0006-3223(00)01088-x

[39] Raison CL Borisov AS Woolwine BJ Interferon-alpha effects on diurnal hypothalamic-pituitary-adrenal axis activity: relationship with proinflammatory cytokines and behavior. *Mol Psychiatry* 2010; 15:535–547. 18521089 10.1038/mp.2008.58 PMC3403676

[40] Thomas MK Lloyd-Jones DM Thadhani RI Hypovitaminosis D in medical inpatients. *N Engl J Med* 1998; 338:777–783. 9504937 10.1056/NEJM199803193381201

[41] Lucas SM Rothwell NJ Gibson RM The role of inflammation in CNS injury and disease. *Br J Pharmacol* 2006; 147 Suppl 1:S232–240. 16402109 10.1038/sj.bjp.0706400 PMC1760754

[42] Allan SM Rothwell NJ Inflammation in central nervous system injury. *Philos Trans R Soc Lond B Biol Sci* 2003; 358:1669–1677. 14561325 10.1098/rstb.2003.1358 PMC1693261

[43] Sayeed I Stein DG Progesterone as a neuroprotective factor in traumatic and ischemic brain injury. *Prog Brain Res* 2009; 175:219–237. 19660659 10.1016/S0079-6123(09)17515-5

[44] Bernd P The role of neurotrophins during early development. *Gene Expr* 2008; 14:241–250. 19110723 10.3727/105221608786883799 PMC6042000

[45] Neveu I Naveilhan P Baudet C 1,25-dihydroxyvitamin D3 regulates NT-3, NT-4 but not BDNF mRNA in astrocytes. *Neuroreport* 1994; 6:124–126. 7703399 10.1097/00001756-199412300-00032

[46] Rose CR Blum R Pichler B Truncated TrkB-T1 mediates neurotrophin-evoked calcium signalling in glia cells. *Nature* 2003; 426:74–78. 14603320 10.1038/nature01983

[47] Kang H Schuman EM Long-lasting neurotrophin-induced enhancement of synaptic transmission in the adult hippocampus. *Science* 1995; 267:1658–1662. 7886457 10.1126/science.7886457

[48] Leffa DD Valvassori SS Varela RB Effects of palatable cafeteria diet on cognitive and noncognitive behaviors and brain neurotrophins’ levels in mice. *Metab Brain Dis* 2015; 30:1073–1082. 25998605 10.1007/s11011-015-9682-0

[49] Eyles DW Smith S Kinobe R Distribution of the vitamin D receptor and 1α-hydroxylase in human brain. *J Chem Neuroanat* 2005; 29:21–30. 15589699 10.1016/j.jchemneu.2004.08.006

[50] Kalueff AV Lou Y-R Laaksi I Increased anxiety in mice lacking vitamin D receptor gene. *Neuroreport* 2004; 15:1271–1274. 15167547 10.1097/01.wnr.0000129370.04248.92

[51] Kim JT Park MS Yoon GJ White matter hyperintensity as a factor associated with delayed mood disorders in patients with acute ischemic stroke. *Eur Neurol* 2011; 66:343–349. 22095210 10.1159/000332585

